# Effect of Different Sealers and Irrigants on the Push-Out Bond Strength of Fiber Posts to The Root Canal

**DOI:** 10.34172/joddd.44101

**Published:** 2026-03-30

**Authors:** Funda Fundaoğlu Küçükekenci

**Affiliations:** Department of Endodontics, Faculty of Dentistry, Ordu University, Ordu, Türkiye

**Keywords:** Chitosan, Dental cements, Dentin-bonding agents, Post-and-core technique, Root canal sealers

## Abstract

**Introduction:**

This study evaluated the effects of bioceramic and resin-based root canal sealers and different irrigation protocols on the push-out bond strength (PBS) of fiber posts.

**Methods:**

Fifty-four freshly extracted mandibular canines were instrumented and irrigated with NaOCl and EDTA. The specimens were first divided into two groups based on the sealer used: a bioceramic sealer (Bioserra) or a resin-based sealer (AH Plus) (n=27 per group). Each group was then randomly subdivided into three subgroups (n=9) based on the irrigation solution used for smear layer removal during post space preparation: distilled water, 17% EDTA, or 0.2% chitosan. Fiber posts were cemented with self-adhesive resin cement. Sections (1.5-mm thick) from the coronal third were subjected to a push-out bond strength test. Data was analyzed using two-way ANOVA and post hoc Bonferroni tests (α=0.05).

**Results:**

Both the type of sealer and the irrigation protocol significantly influenced PBS values (*P*<0.05). The highest PBS was observed in the bioceramic sealer+chitosan group (16.08±0.48 MPa), whereas the lowest value was recorded in the bioceramic sealer+distilled water group (11.20±0.58 MPa).

**Conclusion:**

Root canal sealer type and irrigation protocol during post space preparation significantly affected the bond strength of fiber posts. Chitosan irrigation demonstrated superior performance, suggesting its potential to improve post retention.

## Introduction

 Post-endodontic restoration significantly impacts the prognosis of the treated tooth. An adequate restoration can prevent fractures and support the longevity of the tooth, thereby enhancing treatment outcomes.^1^ Previous studies have indicated that the primary reasons for the extraction of endodontically treated teeth include periodontal disease, endodontic failure, and non-restorable damage due to fractures or caries.^2,3^ Choosing an appropriate restoration is essential for compensating for the loss of coronal tooth structure and ensuring the success of post-endodontic restorations.^1^ Adequate restoration following endodontic treatment is essential, especially when there is insufficient remaining tooth structure; commonly accepted methods include using fiber posts to enhance retention and support for the final restoration. Fiber posts provide several advantages over traditional metal posts, including improved aesthetics, better stress distribution, and compatibility with tooth structure.^4,5^ The bond strength of fiber posts to root dentin is a critical determinant of clinical success. Insufficient adhesion may lead to post-debonding, microleakage, secondary caries, and eventual restoration failure.^6^ Debonding at the cement–dentin interface has been reported as one of the most common failure modes of fiber post restorations, followed by post fracture and root fracture.^7^ Therefore, optimizing the bonding interface between the fiber post, resin cement, and root dentin is essential to enhance the longevity of post-endodontic restorations.^8^ However, the effectiveness of fiber posts depends on various factors that influence their bond strength to dentin. These factors include the quality of adhesion between the post and the root canal wall, the type of material selected for the post, and the adhesive system used during luting.^6^ Additionally, the choice of endodontic sealers and restorative materials can significantly affect the bond strength of fiber posts.^7,8^ Research indicates that the type of root canal sealer used, particularly the differences between resin-based and bioceramic sealers, can markedly impact post retention.^9,10^

 During post space preparation, a smear layer develops due to dentin preparation, potentially acting as a barrier to effective bonding. This layer, consisting of debris and denatured collagen fibers, obstructs adhesive penetration, thereby compromising the bond strength of fiber posts.^11,12^ Therefore, effectively removing the smear layer before cementing a fiber post is crucial, as it can enhance bond strength by enabling deeper penetration of adhesive materials into the dentinal tubules.^13,14^ Chemicals such as ethylenediaminetetraacetic acid (EDTA) and chitosan have been investigated for their effectiveness in smear layer removal. EDTA, a known chelating agent, selectively removes hydroxyapatite and non-collagenous components from dentin, which may improve bond strength.^4,13^ Chitosan, a biopolymer derived from chitin, has shown the potential to enhance the surface quality of dentin and aid better adhesion due to its beneficial chemical properties.^7,14^

 Push-out bond strength (PBS) testing is widely used to evaluate intracanal post adhesion because it provides a more homogeneous distribution of stress along the bonded interface compared with conventional shear or tensile tests. Furthermore, the push-out method allows assessment of bond strength at specific root levels and is considered a reliable and reproducible technique for simulating clinical dislodgement forces.^15,16^

 Although the existing literature notes the efficacy of various irrigation solutions for smear layer removal, a gap remains in research specifically exploring the effect of the interaction between endodontic sealers and post space irrigation solutions on the adhesion of fiber posts.^17,18^ Understanding these interactions is critical for optimizing treatment strategies in endodontics, ultimately aiming to enhance bonding efficiency and clinical outcomes. Thus, this study aims to elucidate the impacts of bioceramic and resin-based sealers on the PBS of fiber posts, specifically assessing the efficacy of EDTA and chitosan in smear layer removal during post space preparation. Our null hypotheses were: (I) There is no effect of sealer type in the PBS of fiber posts cemented with the following canal treatment with bioceramic and resin-based sealers; and (II) The use of distilled water, EDTA, and chitosan does not affect the PBS of fiber posts.

## Methods

 This study was approved by the Ordu University Clinical Research Ethics Committee (Number: 2025/178). The sample size calculation was based on a previous study evaluating the PBS of bioceramic sealers under similar in vitro conditions.^19^ Using the mean and standard deviation values reported in that study, the effect size was calculated. Power analysis was performed using G*Power software (Version 3.1.9.7, Heinrich Heine University, Düsseldorf, Germany) with a significance level of α = 0.05 and a power of 80% (1–β = 0.80), which indicated that a minimum of 9 discs per subgroup was required for the PBS test.

 Fifty-four freshly extracted, single-rooted, caries- and resorption-free, fully developed mandibular canines were used. The presence of a single canal was confirmed using three-angle radiographs. The teeth were stored in 1% thymol solution until used and cleaned of debris with a periodontal curette. All procedures were performed by a single operator to ensure standardization. The teeth were decoronated below the cementoenamel junction using a water-cooled diamond separator, and root lengths were standardized to 12 mm. The working length was determined at 1 mm shorter than the length at which a #15 K-file (Dentsply Sirona, Ballaigues, Switzerland) was visible at the apex. Root canal preparation was performed at the working length using the crown-down technique with a Reciproc Blue R25 (VDW, Munich, Germany) file in reciprocation mode using a VDW Gold endodontic motor (VDW, Munich, Germany). Irrigation was performed with 5 mL of 17% EDTA followed by 5 mL of 2.5% NaOCl for 1 minute each.

 After chemomechanical preparation, the teeth were randomly divided into two main groups (n = 27):

 Group 1: Bioceramic root canal sealer group. The root canals were filled using Bioserra (Bioserra Dentac, Öncü, İstanbul, Türkiye) and gutta-percha with the cold lateral compaction technique.

 Group 2: Resin-based root canal sealer group. The root canals were filled using AH Plus (Dentsply DeTrey, Konstanz, Germany) and gutta-percha with the cold lateral compaction technique.

 Cold lateral compaction was preferred to standardize the obturation technique across groups and eliminate the potential influence of the obturation technique as a confounding variable. After trimming excess gutta-percha, vertical condensation was performed. Root canal orifices were sealed with cotton pellets and restored using Orafil-G (Prevest DenPro Ltd., Jammu, India).

 Bioserra is a hydraulic material that undergoes a hydration reaction and is capable of reaching its initial set within a few hours in the presence of moisture. In contrast, epoxy resin-based materials such as AH Plus exhibit longer polymerization times, with complete setting reported within 24 hours at 37°C. Therefore, although shorter setting periods may be sufficient for bioceramic sealers, selecting a 24-hour setting time in experimental studies ensures complete setting of both calcium silicate- and resin-based sealers and allows for standardized and reliable comparison between groups. Thus, the specimens were stored at 37°C and 100% humidity for 24 hours to allow complete setting of the sealers.^20,21^

 The filling material was removed using a standardized technique. The coronal 3 mm of filling material was removed using Gates-Glidden burs #4 (Dentsply Maillefer, Ballaigues, Switzerland). The final post space preparation was completed using a blue post drill (Reforpost; Angelus, Londrina, PR, Brazil), and a total of 8 mm of filling material was removed.

 Each main group was then subdivided into three subgroups (n = 9) according to the final irrigation protocol:

 Group 1A: 15 mL of distilled water for 3 minutes

 Group 1B: 15 mL of 17% EDTA for 3 minutes

 Group 1C: 15 mL of 0.2% chitosan for 3 minutes

 Group 2A: 15 mL of distilled water for 3 minutes

 Group 2B: 15 mL of 17% EDTA for 3 minutes

 Group 2C: 15 mL of 0.2% chitosan for 3 minutes

 Following EDTA or chitosan irrigation, a final rinse with distilled water was performed to remove residual irrigant and minimize potential interference with resin cement polymerization. To prepare the 0.2% chitosan solution, 0.2 g of chitosan was dissolved in 100 mL of 1% acetic acid. Irrigation procedures were performed using an irrigation needle (Kerr Hawe Irrigation Probe, KerrHawe SA, Biggio, Switzerland). After final irrigation, canals were dried with paper points. The post spaces were filled with a self-adhesive resin cement (RelyX U200, 3M ESPE, St. Paul, MN, USA) using automix tips according to the manufacturer’s instructions. Fiber posts (1.5 mm in diameter; Reforpost, Angelus, Londrina, PR, Brazil) were inserted with mild pressure and polymerized using a light-emitting diode curing unit (Elipar S10, 3M ESPE, Neuss, Germany). All the specimens were stored at 37°C and 100% humidity for 24 hours. Then, the specimens were embedded in acrylic blocks (Meliodent, Bayer Dental, Leverkusen, Germany) and sectioned using a low-speed saw (Mecatome T180, Presi Metallography, Eybens, France). Three 1.5-mm-thick slices were obtained from the coronal third of each root. The second slice was selected for the push-out test.

 Push-out testing was performed using a universal testing machine (Autograph AGS-X, Shimadzu Co., Kyoto, Japan). Load was applied in an apicocoronal direction using a 1-mm diameter plunger at a crosshead speed of 0.5 mm/min until post dislodgement occurred. The maximum load at failure was recorded in Newtons (N) and converted to megapascals (MPa) by dividing by the bonding area (mm^2^). Although root canals exhibit a tapered anatomy, 1.5-mm-thick slices were obtained from the coronal third, where canal taper is relatively limited. In such thin sections, the difference between apical and coronal radii is minimal; therefore, the bonding area was approximated using the cylindrical formula to standardize calculations and ensure methodological consistency with similar push-out bond strength studies.^15,16^ Therefore, the bonding area was calculated using the cylindrical formula π (r₁ + r₂)*h, where h is the slice thickness, r₁ is the apical radius, r₂ is the coronal radius, and π = 3.14.

 Debonded surfaces were examined under a stereomicroscope (Leica SP1600; Leica Microsystems, Wetzlar, Germany) at × 25 magnification. Failure modes were classified as:

1. Adhesive (failure at the dentin–cement or post–cement interface)
2. Mixed (a combination of adhesive and cohesive failure)
3. Cohesive (failure within the resin cement)

Statistical analysis was performed using IBM SPSS Statistics v20.0 (IBM Corp., Armonk, NY, USA). Data normality and homogeneity were confirmed (Levene’s test, *P* = 0.173). PBS values were analyzed using two-way ANOVA to evaluate the effects of sealer type, irrigation solution, and their interaction. Pairwise comparisons were conducted using post hoc Bonferroni tests (α = 0.05).

## Results

 According to the two-way ANOVA results, irrigation solution, sealer type, and the interaction between these factors significantly affected the PBS values (*P* < 0.001) (Table 1).

**Table 1:** Two-way ANOVA results of PBS values

**Source**	**SS**	**df**	**MS**	**F**	***P***
Sealer Type (A)	1.602	1	1.602	562.628	0.000
Irrigation Solution (B)	177.343	2	88.672	31148.269	0.000
A X B	2.843	2	1.422	499.383	0.000
Error	.137	48	0.003		
Total	10140.410	54			

*P < 0.05 indicates a significant difference.

 The mean ± standard deviation (SD) values and the results of post hoc Bonferroni comparisons are presented in Table 2. The highest PBS value was observed in group 1C (Bioserra + chitosan) (16.08 ± 0.48 MPa), whereas the lowest PBS value was observed in group 1A (Bioserra + distilled Water) (11.20 ± 0.58 MPa).

**Table 2:** The means, standard deviations (SD), and post hoc Bonferroni test results of PBS values of test groups

**Groups**		**PBS**
**Sealer type**	**Irrigation solution**	
Bioserra	DW	11.20 (0.58)^Aa^
	EDTA	13.97 (0.29)^Bb^
	Chitosan	16.08 (0.48)^Bc^
AH Plus	DW	11.50 (0.46)^Ba^
	EDTA	13.23 (0.22)^Ab^
	Chitosan	15.50 (0.88)^Ac^

* Post hoc Bonferroni tests of PBS values are presented as superscripts, and significant differences are indicated with different letters (*P* < 0.05). While the superscript with upper case letters indicates the comparisons of different sealer material groups irrigated with solutions

 Within each sealer group, EDTA and chitosan irrigation resulted in significantly higher PBS values compared with distilled water (*P* < 0.05). Chitosan showed significantly higher bond strength than EDTA in both sealer groups (*P* < 0.05).

 Regarding comparisons between sealer types under the same irrigation protocol, AH Plus demonstrated significantly higher PBS values than Bioserra in the distilled water group, whereas Bioserra exhibited significantly higher values in the EDTA and chitosan groups (*P* < 0.05) (Table 2).

 Intergroup comparisons are summarized in Table 2, where different uppercase letters indicate significant differences between sealer types within the same irrigation protocol, and different lowercase letters indicate significant differences among irrigation solutions within the same sealer group (*P* < 0.05). The distribution of failure modes is presented in Table 3. The most common failure mode was mixed (51.85%), followed by adhesive failure (35.19%) and cohesive failure (12.96%).

**Table 3:** Type of failure modes in groups

**Groups**		**Adhesive**	**Cohesive**	**Mixed**	**Total**
**Sealer type**	**Irrigation solution**				
Bioserra	DW	4 (44.44%)	1 (11.11%)	4 (44.44%)	9
	EDTA	3 (33.3%)	1 (11.11%)	5 (55.6%)	9
	Chitosan	2 (22.22%)	1 (11.11%)	6 (66.67%)	9
AH Plus	DW	4 (44.44%)	1 (11.11%)	4 (44.44%)	9
	EDTA	4 (44.44%)	1 (11.11%)	4 (44.44%)	9
	Chitosan	2 (22.22%)	2 (22.22%)	5 (55.56%)	9
Total		19 (35.19%)	7 (12.96%)	28 (51.85%)	54

## Discussion

 The present study showed that both the type of root canal sealer used during canal obturation and the irrigation solution employed during post space preparation significantly influenced the PBS of fiber posts. Therefore, the null hypotheses were rejected. The highest PBS values were observed in the group where chitosan was utilized during post space preparation following obturation with a bioceramic sealer. In contrast, the lowest PBS values were observed in the group where distilled water was used. Furthermore, groups irrigated with chitosan exhibited greater PBS values than those treated with EDTA. The lowest PBS values were consistently observed in the groups where distilled water was the chosen irrigation solution for both sealer types.

 An important consideration is the clinical relevance of the coronal third, as restoration efforts predominantly address the coronal third of the teeth, which endures significant occlusal and functional forces during routine mastication. Previous literature indicates that the coronal third typically exhibits distinct mechanical properties compared to apical regions, underscoring the need to assess bond strength behaviors distinctly.^16,22^ Additionally, studies have highlighted that the adhesive performance and failure loads within the coronal third differ from those observed in the middle or apical thirds.^15,22,23^ The coronal third often exhibits superior bond strengths.^16,22^ This situation can be attributed to the greater thickness of dentin in the coronal third and the presence of more favorable bonding interfaces compared to the apical and middle thirds, thereby enhancing the likelihood of successful bonding in clinical applications.Middle and apical thirds were not analyzed in this study due to their distinct dentin structure and bonding characteristics, which could introduce variability less relevant to the clinical evaluation of coronal restorations.^16,23^

 Thus, the selective examination of the coronal third for PBS not only aligns with clinical necessities but also enhances the understanding of specific adhesive properties relevant to restorative dentistry.^19,24^ Therefore, by concentrating exclusively on the coronal third, the study aims to yield results that have high clinical applicability and relevance, allowing for better interpretation in terms of restoration longevity and efficacy.^16,23^ Thus, samples taken from the coronal third of the root were used for the PBS test in the present study.

 The varying effects of bioceramic sealers on the bond strength of fiber posts may stem from differences in their physicochemical characteristics and their interaction with root dentin. Fiber post retention largely depends on effective adhesion between resin cement and the intraradicular dentin surface.^7^ Residual root canal sealer on dentinal walls may therefore influence the bonding performance of fiber posts.^25^ Bioceramic sealers have been reported to exhibit superior biocompatibility and bioactive properties, potentially promoting chemical interaction with dentin.^26^ In contrast, epoxy resin-based sealers primarily provide mechanical interlocking due to their resin matrix but lack bioactivity that may enhance chemical bonding.^27^ These differences in sealer composition may alter the interfacial characteristics between dentin, resin cement, and fiber posts, thereby affecting push-out bond strength values. Despite the use of post space preparation procedures, complete removal of root canal sealer remnants from dentinal walls is unlikely.^28^ Residual sealers may alter dentin surface energy, wettability, and micromechanical retention, thereby influencing the adhesion of resin cement and ultimately affecting fiber post retention.^7,28^ Epoxy resin-based sealer remnants may demonstrate partial chemical compatibility with resin-based luting cements due to their resin matrix, potentially allowing limited micromechanical interlocking or interfacial adaptation.^29^ However, once polymerized, epoxy residues may also function as a weak intermediate layer and compromise bonding if inadequately removed.^30^ In contrast, bioceramic sealer remnants are hydrophilic and calcium silicate-based. However, they exhibit bioactivity and chemical interaction with dentin; they lack methacrylate components necessary for copolymerization with resin cements, which may negatively influence adhesive performance.^26^

 Chelating agents such as EDTA primarily interact with calcium ions and are therefore more effective in modifying or partially dissolving calcium silicate-based (bioceramic) remnants.^31^ Nevertheless, their effect is not limited exclusively to bioceramic materials. By removing the smear layer and altering dentin permeability and surface morphology, chelating agents may also indirectly influence the bonding environment of resin-based sealers and resin cement.^32^

 Irrigation solutions play a crucial role in determining the bond strength of fiber posts after root canal obturation. They directly influence the removal of the smear layer and debris that form during root canal preparation.^11,12^ EDTA is widely recognized for its ability to effectively remove the smear layer, thereby enhancing bonding. The use of EDTA has been shown to lead to significant alterations in the dentin surface, as it removes the smear layer and exposes the dentinal tubules, enhancing the overall cleanliness and disinfection of the root canal.^33-35^

 Studies demonstrate that appropriate concentration and exposure time are crucial to maximize the positive effects while minimizing adverse impacts on dentin integrity.^25,36^ The optimal protocol is often cited as 17% EDTA applied for a few minutes to ensure efficient removal of debris without severely compromising dentin microhardness.^26^ Many studies have shown that a duration of 3 minutes is frequently optimal for achieving sufficient smear layer removal without significant erosive effects on dentin.^25-27^ Dorileo et al.^37^ showed that applying chitosan results in favorable bonding, even compared to EDTA. This situation suggests that chitosan might not only sufficiently clear the smear layer but also provide additional benefits, such as enhancing dentin collagen integrity and supporting interfacial adhesion due to its chemical composition. In addition, Penumaka et al.^38^ reported that chitosan could enhance the removal efficacy compared to EDTA alone while also being less erosive to dentin structures. This dual function renders chitosan a valuable adjunct to EDTA in endodontic irrigants. Several studies have indicated that a duration of approximately 3 minutes is effective for the application of chitosan as a final irrigation solution to achieve optimal smear layer removal. In this time frame, chitosan not only facilitates the removal of debris but also enhances the overall cleansing of the dentin surface.^39,40^ Thus, the application time of EDTA and chitosan was determined as 3 minutes in the present study.

 Moreover, the improved PBS observed with chitosan may be attributed to its characteristics as a natural biopolymer. Chitosan aids in the creation of a cleaner dentin surface while maintaining the integrity of collagen fibers, thus facilitating greater sealer penetration into dentinal tubules.^39^ On the other hand, while EDTA successfully eliminates the smear layer, it does so at the expense of exposing collagen, which may subsequently lead to a weaker bond under certain conditions.^41,42^

 Although chitosan-treated groups demonstrated the highest PBS values overall, EDTA-treated samples still exhibited higher bond strength than the distilled water group. This finding highlights that effective smear layer removal, regardless of the chelating agent used, is essential for improving fiber post adhesion, whereas distilled water lacks sufficient surface-modifying capability. Despite the lower PBS values associated with groups treated with chitosan, the bond strengths exhibited by EDTA-treated samples were still higher than those observed in the distilled water group. This finding implies that while distilled water is ineffective for smear layer removal, it does not provide the enhanced bonding environment fostered by more effective irrigants.^43^ As such, samples treated with distilled water after filling displayed inadequate bonding capacity, potentially due to the distilled water’s insufficient ability to effectively clean the surface or disrupt the adhesive layers necessary for a robust bond.^44^ According to these results, it is plausible that the diminished bond strength observed in distilled water groups stems from the inadequate removal of bioceramic sealer residues, leading to compromised adhesion. Moreover, the improved PBS observed with chitosan may be attributed to its characteristics as a natural biopolymer. Chitosan facilitates effective smear layer removal while preserving collagen integrity, which may enhance micromechanical interlocking and resin penetration into dentinal tubules.^39,40^ In contrast, although EDTA effectively removes the smear layer, prolonged or excessive demineralization may lead to collagen exposure and potential structural weakening of the dentin substrate. ^45^

 In the present study, chitosan-treated groups demonstrated higher PBS values compared to EDTA-treated groups. This finding suggests that the more controlled and less aggressive chelating action of chitosan may create a more favorable bonding substrate for fiber post adhesion. ^32,46^

 This indicates that effective smear layer removal—regardless of the agent used—is critical for optimizing adhesion. Distilled water, which lacks chelating properties, is insufficient to modify the dentin surface adequately, which may explain the consistently lower PBS values observed in those groups. An additional point that should be considered when interpreting the findings of the present study is the potential influence of the solvent used for chitosan preparation. Chitosan is typically dissolved in diluted acetic acid, and weak organic acids may cause mild dentin demineralization and partial smear layer modification. Therefore, it could be argued that part of the observed effect might be associated with the solvent rather than the chelating action of chitosan itself. However, previous research comparing chitosan with acetic acid alone has shown that chitosan exhibits significantly greater smear-layer removal capacity. For instance, Silva et al.^39^ reported that 0.2% chitosan was more effective than 1% acetic acid in removing the smear layer after root canal instrumentation. This finding suggests that the dentin-modifying effect of the chitosan solution is not solely attributable to the acetic acid solvent but is largely related to the intrinsic chelating properties of chitosan. Nevertheless, future studies including a control group treated with acetic acid at the same concentration and pH as the chitosan solution would help further clarify the relative contribution of the solvent.

 It is crucial to acknowledge the limitations of this study. Primarily, the evaluation of PBS values was conducted under conditions subjected solely to vertical forces. However, in clinical scenarios, post-restored teeth are often exposed to multiple force vectors, necessitating comprehensive research strategies that consider varying loads and directional forces.^47^ Another limitation of the present study is that only the coronal third of the root was evaluated. Although this region is clinically relevant due to its exposure to higher functional loads and its importance in restorative procedures, the middle and apical thirds were not assessed. Since dentin structure, tubule density, and bonding characteristics vary along the root canal, the push-out bond strength values obtained from the coronal third may not fully represent the behavior of fiber posts in other root regions. Future studies incorporating middle and apical sections would provide a more comprehensive understanding of regional differences in bond strength. Understanding the interaction of different force directions on bond strength could offer significant insights into the material performance in vivo. Additionally, studies examining the interactive effects of bioceramic and epoxy resin-based sealants alongside various irrigation solutions on PBS are scant in the literature. No studies were found that explicitly evaluated the interactions between specific bioceramic sealants and different irrigation solutions on fiber post bond strength. Future clinical studies that compare the results of the present study with clinical practices will enrich our understanding of the clinical relevance and applicability of these results.

## Conclusion

 The present study showed that both the type of root canal sealer and the irrigation solution used during post space preparation significantly influenced the PBS of fiber posts. Specifically, the results indicated that using chitosan as an irrigation solution after filling with bioceramic sealer yielded superior PBS compared to distilled water and EDTA.

## Competing Interests

 The author declares that she has no known competing financial interests or personal relationships that could have influenced the work reported in this paper.

## Ethical Approval

 This study was approved by Ordu University Clinical Research Ethics Committee (178/2025).
